# Clinically Anchored GPCR Drug Discovery: Translational Lessons from Success, Failure, and Post-Approval Reassessment

**DOI:** 10.3390/ph19101510

**Published:** 2026-09-23

**Authors:** Yohan Seo, Joohan Woo

**Affiliations:** 1 Department of Physiology, College of Medicine, Dongguk University, Gyeongju 38066, Republic of Korea; 2 Medical Cannabis Research Center, College of Medicine, Dongguk University Wise, 32 Dongguk-ro, Ilsan Dong-gu, Goyang 10326, Republic of Korea

**Keywords:** G protein-coupled receptor, translational pharmacology, drug modality, pharmacokinetics/pharmacodynamics, biased agonism, chemokine receptor, sphingosine-1-phosphate receptor, complement C5a receptor, rare disease, data integrity

## Abstract

**Background/Objectives:** G protein-coupled receptors are a productive drug target family, yet clinical performance varies widely across rare, immune-mediated, pain, and thromboinflammatory diseases. We aimed to construct an explicit, stage-gated framework that makes translational prioritisation auditable and to examine whether it distinguishes clinically successful from unsuccessful programmes. **Methods:** This narrative, clinically anchored review included programmes with a stated human-disease rationale, Phase 2 or later clinical experience or a regulatory decision, and publicly accessible primary or regulatory documentation; evidence was considered through 7 August 2026. The cases spanned rare disease, immune-mediated inflammation, pain, and thromboinflammation. Thirteen programmes were reviewed, and eleven were entered into a retrospective five-axis scorecard covering human-disease anchoring, receptor pharmacology, modality and pharmacokinetic/pharmacodynamic feasibility, biomarker-to-endpoint readiness, and durability. Each axis was scored 0–2, weighted to 100 points, and subject to predefined hard-stop rules. **Results:** Established successes scored 80–100 points, whereas failed programmes scored 30–48. PAR4 scored 57.5 and therefore fell in the stop-and-rework band; avacopan scored 80 before approval but now triggers an Axis 5 hard stop. Equal weighting preserved the classifications of established successes and hard-stop failures but shifted PAR4 to 60.0, demonstrating sensitivity of a borderline programme to the weighting scheme. The scorecard localized failures mainly to redundancy, biomarker–endpoint disconnect, or durability/evidence-integrity gates. **Conclusions:** The framework is an explicit prioritisation aid, not an externally validated predictive model. Its main value is to identify missing evidence and non-compensable weaknesses; prospective independent validation, including inter-rater reliability testing, is required before portfolio use.

## 1. Introduction

Whether G protein-coupled receptors (GPCRs) are druggable is no longer in question: they are the targets of roughly one-third of approved medicines, with 475 drugs acting on 108 receptors reported in 2017 and 516 drugs acting on 121 receptors in the 2025 update [1,2]. The pressing question for pharmaceutical science is narrower and more challenging: which GPCRs are translationally de-risked sufficiently, and through which modality and exposure strategy, to justify development for a specific disease [3,4,5]? A receptor may be structurally ligandable, highly expressed in diseased tissue, and tractable in vitro, yet still fail if the target–disease link is redundant, the pharmacodynamic (PD) biomarker is weak, the clinical endpoint is mismatched, the exposure–response relationship is unfavorable, or the safety window is too narrow.

This review is not an encyclopedic survey. Instead, it examines programs that reached patients and yielded clear translational lessons through success, failure, or regulatory reassessment. We give particular attention to modality and pharmacokinetic/pharmacodynamic (PK/PD) because the choice among a small molecule, peptide, and biologic determines exposure, route of administration, reversibility, tissue access, target engagement, and the practical management of adverse effects.

Section 2, Section 3 and Section 4 present the clinically anchored case analyses as Results. Section 5 discusses prospective applications, limitations, and formulation implications. Section 6 describes the derivation, scoring, and application of the five-axis framework, and Section 7 summarizes the main conclusions.

### Scope and Case-Selection Criteria

This is a narrative, clinically anchored review rather than a systematic review or meta-analysis, and the case set was fixed against criteria defined before the analysis began. A programme was included only if it satisfied all three of the following conditions: (i) the GPCR had a stated human-disease rationale at the time the programme was nominated; (ii) the programme had reached Phase 2 or later, or a regulatory decision, so that a clinical outcome rather than a preclinical projection was available; and (iii) primary trial publications or regulatory documents were publicly accessible, so that every claim made here is traceable to a source rather than to a secondary summary.

Programmes were excluded when the clinical outcome was still pending, when the only available evidence was a conference abstract or a company announcement, or when development stopped for reasons unrelated to receptor pharmacology, such as portfolio or financing decisions. The last exclusion matters for interpretation: a programme discontinued on commercial grounds carries no translational lesson, and counting it as a pharmacological failure would misstate how the receptor class has actually performed.

Applying these criteria to records retrieved from PubMed, ClinicalTrials.gov, and FDA and EMA documents considered through 7 August 2026 produced thirteen clinically anchored programmes across four domains: rare disease, immune-mediated inflammation, pain, and thromboinflammation. Eleven programmes were entered into the retrospective scorecard presented in Section 6.7; avexitide and oliceridine were retained as qualitative comparator cases for modality and signaling concepts rather than included in the score-separation analysis. For each scored programme we extracted the human-disease evidence, receptor pharmacology, modality and PK/PD profile, target-engagement or pharmacodynamic biomarker, clinical endpoint, safety findings, and evidence reliability.

The selection pathway is shown below. Records from PubMed, ClinicalTrials.gov, FDA, and EMA sources were screened against the three eligibility criteria, yielding thirteen programmes for narrative review and eleven for the quantitative scorecard. Because this was a narrative review and deduplicated screening counts were not prospectively logged, numerical exclusion totals are not reconstructed retrospectively.

**Selection flow:** PubMed + ClinicalTrials.gov + FDA/EMA records (initial count not prospectively enumerated) → eligibility criteria applied → 13 clinically anchored programmes → 11 scorecard programmes + 2 qualitative comparators

The resulting set is curated and is therefore vulnerable to selection, survivorship, and hindsight bias, and no pooled effect estimate and no prospective predictive accuracy is claimed. What the set does permit is a like-for-like comparison of why programmes with comparable receptor rationale diverged in clinical outcome.

Figure 1 summarizes the clinically anchored GPCR programs selected for this review and links each therapeutic domain to its corresponding evidence tier and translational lesson.

Score key (weighted totals; see Section 6.7): CXCR4–WHIM 92.5; MC4R–obesity 82.5; S1PR–UC 80.0; CGRP–migraine 100; C5aR1–AAV 72.5 *; PAR1–thrombosis 82.5; PAR4–thrombosis 57.5; CCR9–Crohn disease 30.0*; DP2–asthma 47.5 *; CCR2/CCR5–NASH 37.5 *; GPR84–fibrosis 25.0 *. * Hard-stop rule overrides the numerical total.

## 2. Results: Rare-Disease GPCRs with Strong Human-Disease Anchoring

The scoring procedure, stage-specific hard stops, weighting scheme, and operational score anchors are described in Section 6 and Supplementary Table S1.

Rare-disease GPCR programs succeed disproportionately when the receptor lies on the causal pathway of a monogenic or anatomically defined disorder. The target–disease relationship is then established by human genetics or a defined lesion rather than inferred from expression, and the same causal biology usually provides a quantitative PD readout.

### 2.1. CXCR4–Mavorixafor in WHIM Syndrome

WHIM syndrome is caused by gain-of-function alterations in the CXCR4 C-terminal region that impair receptor internalization, increase CXCL12/CXCR4 signaling, and retain leukocytes in the bone marrow [6,7,8]. In the 4WHIM trial, mavorixafor increased the time above threshold for absolute neutrophil and lymphocyte counts and reduced infection measures, although warts remained unchanged [9,10,11,12,13]. The mechanism-to-endpoint chain is explicit: genotype defines the population, receptor blockade changes absolute neutrophil and lymphocyte counts (ANC/ALC) in real time, and these counts are linked to infection risk. The program scores 92.5/100; the remaining uncertainties concern the small sample size and long-term manifestations that are not expected to respond fully to CXCR4 blockade.

### 2.2. MC4R–Setmelanotide in Genetic and Acquired Hypothalamic Obesity

MC4R lies downstream of the leptin–POMC pathway. Setmelanotide is effective in selected patients with POMC, PCSK1, or LEPR deficiency and in those with Bardet–Biedl syndrome, and its indication was extended in March 2026 to acquired hypothalamic obesity in patients aged 4 years and older [14,15,16,17]. The human anchor is genetic or anatomic, whereas the main Axis 3 constraint is once-daily subcutaneous administration. The program scores 82.5/100 because strong anchoring and endpoint readiness compensate for, but do not eliminate, route of administration and safety-monitoring constraints.

### 2.3. GLP-1 Receptor Antagonism in Post-Bariatric Hypoglycemia

As a pharmacological counterpoint, GLP-1 receptor *antagonism* (e.g., avexitide/exendin-9-39) is rationally anchored in post-bariatric hyperinsulinemic hypoglycemia, in which exaggerated GLP-1 signaling drives the phenotype and GLP-1 receptor blockade corrects postprandial hypoglycemia [18]. The peptide antagonist avexitide (exendin 9-39) reduced hypoglycemic events in a randomized crossover Phase 2 trial (PREVENT) but has not yet been approved [19].

### 2.4. Cross-Cutting Principles

The rare-disease cases support three operational rules. First, causal genetics or a defined lesion increases the Axis 1 score and clarifies patient selection. Second, the causal mechanism should yield a measurable PD response that can be related to dose and clinical endpoint. Third, the therapeutic indication should remain aligned with the genotype or lesion that provides the causal evidence.

## 3. Results: Immune-Trafficking and Inflammatory GPCRs—When Receptor Biology Does and Does Not Translate

Immune-inflammatory GPCRs are difficult to translate because chemokine and lipid receptor networks are redundant and context dependent. The framework therefore asks whether the selected receptor is nonredundant in the intended population, whether a PD biomarker reflects the relevant pathogenic cellular process, and whether that biomarker predicts the clinical endpoint.

### 3.1. S1P Receptor Modulators—The Positive Case

S1P modulators provide a positive example because receptor internalization, peripheral lymphocyte reduction, and clinical dosing are linked. Ozanimod and etrasimod are approved for ulcerative colitis, although remission rates are modest and class-specific cardiac, ocular, hepatic, and infection-related risks require monitoring [20,21,22,23,24,25,26,27,28]. The program scores 80/100: Axis 1 is not based on a monogenic disease, but strong receptor pharmacology, reversible PD, and a direct lymphocyte readout support translation. Peripheral lymphocyte count should still be viewed as a target engagement biomarker rather than a perfect surrogate for remission.

### 3.2. C5aR1–Avacopan—Approval Is Not Durable De-Risking

Avacopan was approved as an adjunctive treatment for ANCA-associated vasculitis based on the ADVOCATE trial [29]. Its post-approval reassessment now involves two distinct issues. The FDA reported serious DILI, including fatal cases and VBDS [30]. Separately, the pivotal publication was retracted after undisclosed post-unblinding endpoint readjudication, and the EMA recommended revocation because the evidence of effectiveness could no longer be considered reliable; the FDA proposed withdrawal of approval in April 2026 and the European Commission adopted a decision revoking the EU marketing authorisation on 4 August 2026 [31,32]. We therefore record 5A safety and 5B evidence reliability separately. The evidence reliability failure is not evidence against C5aR1 biology; it is a regulatory and governance hard stop that requires independent regeneration of the efficacy evidence.

Figure 2 separates the avacopan post-approval stress test into safety signal and evidentiary integrity tracks because these two failure modes require different interpretations.

### 3.3. CCR9–Vercirnon—Failure by Redundancy

Vercirnon, an oral CCR9 antagonist, blocked CCL25–CCR9-dependent lymphocyte homing to the small intestine. Phase 2 (PROTECT-1) was positive and the Phase 3 SHIELD programme was not, and it is the discordance rather than the negative result that is informative [33,34]. At least three explanations are compatible with the published data. The first is target redundancy: CCR9 is one of several routes by which effector lymphocytes reach inflamed gut, and its blockade may simply be compensated. The second is insufficient tissue engagement: an orally tolerated antagonist need not achieve sustained receptor occupancy in the mucosa. The third is that the Phase 2 estimate was not stable.

The evidence discriminates only partially. Redundancy is the best-supported explanation because the same anatomical process was drugged successfully at a different node: α4β7 blockade with vedolizumab produces durable benefit in the same tissue and the same disease [35,36,37]. Gut homing is therefore a tractable target class, and the failure is specific to the CCR9 node rather than to the trafficking hypothesis. What the programme cannot exclude is the second explanation, because no mucosal occupancy or trafficking biomarker was reported at any stage; in the published record, “engaged but compensated” and “insufficiently engaged” remain observationally equivalent.

This is why the scorecard records 0 on Axis 2 and 0 on Axis 4 rather than a single cause. The Axis 4 zero is the operative hard stop, but it marks the weakest documented link in the evidence chain, not a demonstrated mechanism of failure. A programme returning to this target would need paired mucosal sampling establishing receptor occupancy, and a trafficking readout that moves with it, before an outcome trial rather than after one.

### 3.4. DP2/CRTH2–Fevipiprant—Biomarker Is Not Endpoint

Fevipiprant, an oral DP2 antagonist, lowered sputum eosinophils in Phase 2, but neither LUSTER-1/2 nor ZEAL delivered the intended reduction in exacerbations, and the LUSTER result did not survive adjustment for multiplicity [38,39,40]. Describing this as “biomarker is not endpoint” understates the problem, because two materially different situations generate that pattern and they call for different remedies.

In the first, the biomarker is disconnected. DP2-driven eosinophil recruitment is one input into a type 2 inflammatory network in which IL-5 and IL-4/IL-13 signalling can sustain the exacerbation phenotype, so moving the eosinophil count moves a marker that no longer constrains the outcome. In the second, the biomarker is connected but the drug effect on the endpoint is genuinely small, and the programme was sized and analysed for a larger one; a directionally consistent result that fails after multiplicity adjustment is compatible with this.

The distinction is not academic. If the biomarker is disconnected, enrichment cannot recover the programme and the target requires a different endpoint hypothesis. If the effect is real but small, enrichment for patients in whom DP2 activity is not already covered by the IL-5 and IL-4/IL-13 axes could in principle recover it, and the failure is one of population selection rather than of mechanism. The published programme does not separate the two, and that is itself the finding: Phase 3 was committed before the biomarker had been shown to carry endpoint-predictive information beyond the pathways that approved biologics already address. Axis 4 is scored 0 on that basis.

### 3.5. CCR2/CCR5–Cenicriviroc—Surrogate Is Not Outcome

Cenicriviroc, a dual CCR2/CCR5 antagonist, produced an antifibrotic signal in the Phase 2b CENTAUR study; the Phase 3 AURORA study was terminated for lack of efficacy [41,42,43]. Three explanations remain open on the published evidence, and the programme was not designed to separate them.

The first is that the surrogate does not track the outcome, so that histological improvement at one year did not predict the clinical result. The second is that the Phase 2b signal did not represent a durable drug effect. The third is that hepatic exposure was insufficient to sustain dual receptor blockade at the site of disease; this cannot be excluded, because neither liver target engagement nor an exposure–response relationship was established.

The framework records the failure at Axis 4 with an Axis 3 constraint, and the basis for that assignment should be stated precisely. It is not a claim that macrophage-directed chemokine blockade is biologically wrong in this disease. It is a statement that the programme’s own evidence chain broke where an intermediate readout was used to justify an outcome trial, and that a second link—tissue exposure—was never instrumented, so the failure cannot be attributed with confidence to the mechanism rather than to its delivery. A revived programme would have to establish the exposure–response relationship first, because without it a negative outcome trial cannot distinguish a wrong target from an untested one.

### 3.6. GPR84 and Emerging Macrophage GPCRs

GPR84 antagonism (GLPG1205) reached Phase 2 in ulcerative colitis (program discontinued) and IPF (PINTA, limited efficacy) and was deprioritized [44,45,46]. It belongs in the future directions section; its clinical PD biomarkers remain immature, and its translational readiness should not be overstated.

### 3.7. Decision Rule

The decision rule is prospective. A program should advance only when receptor nonredundancy, target engagement, biomarker-to-endpoint linkage, and a feasible exposure strategy have been demonstrated in the intended population. A low score identifies the missing evidence: CCR9 requires proof of nonredundancy; DP2 and CCR2/CCR5 require endpoint-linked biomarkers; GPR84 requires mature clinical PD biomarkers; and avacopan requires post-approval safety management and independently generated, reliable efficacy evidence.

A general caution follows from these three cases. A scorecard entry localises the weakest documented link in a programme’s evidence chain; it does not adjudicate among the competing mechanisms that could have produced the observed failure. Where a programme never instrumented a link—mucosal occupancy for CCR9, hepatic exposure for cenicriviroc—the framework can record that the link was untested, but it cannot substitute for the missing measurement. Reading a low score as a causal verdict would repeat the inferential error that the framework is intended to prevent.

Figure 3 summarizes this decision rule: whether a programme carries both a causal human-disease anchor and a mechanism-proximal pharmacodynamic readout separates the programmes that translated from those that failed, and a third branch shows that approval itself can be reversed after marketing.

## 4. Results: Cross-Domain Stress Tests—Pain and Thromboinflammation

Pain and thromboinflammation provide a stress test outside the rare-disease and immune-trafficking settings from which the framework was assembled. Each domain contains an approved agent and each loads a different axis: pain asks whether modality choice alone can convert a validated target into a usable medicine, which is an Axis 3 question, whereas thromboinflammation asks whether confirmed target engagement is sufficient when the efficacy–safety window is narrow, which is an Axis 4 and Axis 5A question.

### 4.1. Pain—CGRP as Modality-Defined Validation, and Biased Agonism as a Caution

CGRP-directed migraine therapy shows that one validated receptor axis can support distinct therapeutic modalities. Monoclonal antibodies provide prolonged exposure, monthly dosing, and minimal CYP-mediated interactions, whereas oral gepants provide faster and more reversible exposure for acute or preventive use [47,48,49,50,51,52]. This comparison is useful because target validity is held constant while Axis 3 properties vary.

The μ-opioid receptor illustrates the limitations of signaling bias claims. Oliceridine was developed to improve the separation between analgesia and opioid-related respiratory or gastrointestinal effects, but the improvement in the clinical therapeutic index was limited, and class-related liabilities remained [53,54,55,56,57]. Signaling bias should therefore be scored based on clinically demonstrated benefit–risk rather than on an in vitro bias metric alone.

### 4.2. Thromboinflammation—The Efficacy–Safety Window and the Limits of Target Engagement

Thromboinflammation stresses the safety-window axis. PAR1, the principal thrombin receptor on human platelets, is a validated antithrombotic target: vorapaxar reduced recurrent atherothrombotic events in the TRA 2°P–TIMI 50 trial (26,449 patients) but increased intracranial hemorrhage (1.0% versus 0.5%); therefore, its prescribing information includes a boxed warning for bleeding and contraindications in patients with a prior stroke, transient ischemic attack, or intracranial hemorrhage [58,59,60]. The target is valid, and the biomarker (platelet aggregation) is direct; the primary constraint on the program is the width of the efficacy–safety window—precisely the variable that Axes 3 and 5 are intended to capture.

PAR4 has been pursued as an approach to achieving antithrombotic efficacy with a wider safety margin than PAR1 blockade; candidate antagonists demonstrate human PD target engagement, but no PAR4 agent has completed an outcome trial demonstrating clinical benefit. Therefore, the axis should be scored as target engaged, outcome unproven rather than as de-risked [61,62]. Framing it this way is the core discipline of the rubric: demonstrated target engagement is necessary but not sufficient, and claims of readiness must await clinical endpoint data.

## 5. Discussion

Two findings are most relevant to development decisions. First, mechanism-proximal PD biomarkers facilitate dose selection and early falsification, but they receive a high Axis 4 score only when they are plausibly linked to the clinical endpoint. Second, durability requires separate assessment of pharmacological safety and evidence reliability. The avacopan case demonstrates why a high pre-approval score cannot override a subsequent Axis 5 hard stop.

For formulation and delivery, the key practical consideration is the match between the therapeutic modality and the intended clinical use. Setmelanotide, mavorixafor, etrasimod, avacopan, and erenumab differ in route of administration, exposure duration, reversibility, interaction liability, and monitoring burden. These properties should be specified during target validation rather than treated as downstream formulation considerations.

### 5.1. Prospective Use and Failure Localization

The scorecard is intended to be completed before the next development decision. It does more than rank programs: it identifies the experiment or evidence required to change the decision. A low Axis 2 score calls for receptor selectivity or nonredundancy experiments; a low Axis 3 score calls for a different therapeutic modality or exposure strategy; a low Axis 4 score calls for biomarker mediation, clinical endpoint validation, or improved patient stratification; and a low Axis 5 score calls for safety-risk mitigation or independent verification of the supporting evidence.

The hard-stop rule is the principal distinction between the framework and a list of general success factors. A strong human anchor cannot compensate for an endpoint-disconnected biomarker, and prior approval cannot compensate for unacceptable safety or unreliable pivotal evidence. The score is therefore used to localize failure and guide the next action rather than to create a false impression of mathematical certainty.

### 5.2. Objections and Counterarguments

Four objections can reasonably be raised against a framework of this kind, and each deserves a direct answer rather than a restatement of the framework’s aims.

The first is that scoring rubrics reproduce what experienced developers already know and are assembled once the outcomes are known, so that the cases necessarily fall out correctly. The objection has force, and the retrospective application reported in Section 6.1 cannot refute it. Two features are nevertheless falsifiable. The hard-stop rules make predictions that contradict common practice: a programme with strong human genetics and a feasible modality is normally advanced, whereas here a score of 0 on Axis 4 stops it irrespective of the total. And the framework reports its own failure. Avacopan scored 80 before approval and would have been advanced, because nothing in the pre-approval evidence anticipated the subsequent reversal. The framework localises that failure to Axis 5 in retrospect, but it did not predict it, and a rubric that predicted every outcome in a curated set would warrant more suspicion than one that does not.

The second is that human-disease anchoring is weighted too heavily, given continuing disagreement about how far genetic support improves the probability of clinical success. The 25% weight is not derived from any published estimate of that improvement, and the framework does not depend on a particular effect size. Axis 1 is weighted for its gate function: without a causal human anchor, the population in which efficacy must be demonstrated cannot be defined, which is a structural obstacle rather than a probabilistic one.

The third objection is the most substantial. Evidence reliability is a property of a trial and its conduct, not of a receptor or a molecule, and on that basis, it does not belong on an axis of pharmacological assessment. We accept the premise and reject the conclusion. Axis 5B is deliberately stage-restricted: it is not assessable at nomination, contributes nothing to early scoring, and is applied only once clinical evidence exists. The object being scored, however, is not the molecule but the decision to proceed, and at registration the reliability of the pivotal dataset governs that decision as directly as toxicity does. Avacopan is the demonstration. The receptor biology was not refuted, and the marketing authorisation was withdrawn nonetheless; a framework restricted to pharmacological properties would have scored the programme as sound at the moment it ceased to be available to patients.

The fourth is that the weights and thresholds are arbitrary. They are reasoned rather than fitted, and borderline programmes can be sensitive to them. Under equal weighting, all established successes and hard-stop failures retain their classifications, but PAR4 moves from 57.5 to 60.0 and therefore crosses from stop/rework to conditional/rework. This sensitivity is precisely why the numerical thresholds should be treated as reporting conventions pending validation, whereas the stage-specific hard stops are the more operative component of the framework.

### 5.3. Limitations

Seven limitations should be stated explicitly, and several of them bound what the framework can be used for rather than merely qualifying it.

First, and most importantly, the scoring was performed with knowledge of the clinical outcomes. We did not score the programmes blind, and it is not possible to exclude that knowing an outcome influenced a borderline judgement on an individual axis. The framework should therefore be read as a retrospective explanatory instrument that makes the basis of a judgement explicit and auditable, and not as evidence of prospective predictive accuracy. The two claims are separable: an instrument can organise a known record coherently and still fail to anticipate an unknown one.

Second, the case set is small and curated, and is therefore exposed to selection, survivorship and hindsight bias. Programmes that failed early or silently, and programmes for which primary documentation is unavailable, are systematically under-represented. No pooled estimate and no error rate can be derived from thirteen cases chosen for their instructiveness.

Third, the 0–1–2 scale remains partly subjective. Although the score anchors are operationalized in Section 6.1 and in Supplementary Table S1, assignment of a score to a body of evidence still requires judgement, and we did not perform an inter-rater reliability assessment. A necessary next step is independent rescoring of the same programmes with reporting of weighted kappa and its confidence interval. Poor reproducibility should prompt revision of the score anchors rather than post hoc adjustment of individual cases.

Fourth, the weights and thresholds are reasoned rather than empirically derived, and they have not been externally validated. They were assigned before case scoring on the basis of the expected influence of each axis on a development decision, not fitted to the outcomes. Equal weighting preserves the classifications of established successes and hard-stop failures but moves PAR4 from 57.5 to 60.0, changing this borderline programme from stop/rework to conditional/rework. The thresholds should therefore be read as provisional reporting conventions, and both weights and cut points require prospective validation.

Fifth, non-assessable evidence requires an explicit stage rule. An entire axis is recorded as not assessable (NA) only when the evidence required to score that axis cannot yet exist at the programme’s current stage. A wholly NA axis is excluded from both the weighted numerator and the assessable-weight denominator, and the resulting total is normalized to the weight that is actually assessable; NA is never treated as zero. Axis 5 requires an additional component rule because it contains 5A (safety) and 5B (evidence reliability): if one component is genuinely NA at an early stage, the provisional Axis 5 score is based on the assessable component and is reported explicitly as x/NA. Once both components are assessable, Axis 5 is the lower of 5A and 5B. Conversely, evidence that could reasonably have been generated at that stage but was not measured is scored 0 rather than NA, because the omission itself is a development deficiency.

Sixth, the framework addresses prioritisation and does not address chemistry, manufacturability, intellectual property, or commercial considerations, each of which can determine the fate of a programme independently of the axes considered here.

Seventh, the evidence base has a defined cutoff. The literature and regulatory record were searched to 7 August 2026, and this date applies uniformly to the primary literature, the clinical trial record, and the FDA, EMA and European Commission documents cited, including the avacopan proceedings. Any development after that date is outside the scope of this review.

### 5.4. Implications for Formulation and Delivery

Modality should be considered during target validation because it determines the achievable exposure-response relationship, the reversibility available for safety management, and the monitoring burden incorporated into the prescribing information. Etrasimod illustrates a reversible oral PD profile, erenumab illustrates prolonged exposure with minimal metabolic interactions, and setmelanotide illustrates how the peptide route of administration and receptor-family effects become development constraints. Figure 4 provides a first-pass comparison, but compound-specific data remain necessary.

### 5.5. Future Directions

Three next steps are required. First, the scorecard should be evaluated prospectively by independent reviewers and compared with unweighted and alternative weighting models. Second, emerging macrophage and lipid-sensing GPCRs should advance only when clinical target engagement biomarkers and clinical endpoint linkage have been established. Third, pivotal trials should incorporate predefined, auditable endpoint adjudication procedures consistent with Quality by Design and Good Clinical Practice principles so that evidence reliability is protected throughout development [63,64].

## 6. Materials and Methods: The Five-Axis Translational Framework

The framework is a stage-gated prioritization tool. It combines a weighted 0–2 scoring system with hard-stop rules to ensure that a critical weakness is not concealed by averaging across stronger axes.

### 6.1. Framework Derivation, Scoring and Retrospective Application

The framework is applied as a five-step procedure. The steps are the same whether a programme is being nominated, advanced, or reassessed after approval; only the axes that are assessable change with stage.

Step 1 is to score each axis 0, 1, or 2 against the anchors in Table 1 and to record the evidence used for each score. Step 2 is to check the hard-stop conditions before any arithmetic is performed: a score of 0 on Axis 1, 3, or 4 stops the programme at the corresponding stage irrespective of the total, and from registration onward a score of 0 on either component of Axis 5 does the same. Step 3, if no hard stop applies, is to convert the axis scores into a single total by taking each score as a fraction of its maximum and multiplying by the weight of that axis, so that a programme scoring 2 on every axis reaches 100: total = Σ (score ÷ 2) × weight, with weights of 25% for Axis 1, 15% for Axis 2, 20% for Axis 3, 25% for Axis 4, and 15% for Axis 5. Step 4 is to read the total as advance (≥75), conditional (60–74), or stop and rework (<60), subject to the axis interactions described below. Step 5, which is the output that is actually used, is to record for every axis scoring below 2 the specific experiment or dataset that would raise it.

Vercirnon in Crohn’s disease illustrates the procedure. Its axis scores in the retrospective scorecard (Section 6.7) are 1, 0, 1, 0, and 1, giving a weighted total of (1 ÷ 2) × 25 + (0 ÷ 2) × 15 + (1 ÷ 2) × 20 + (0 ÷ 2) × 25 + (1 ÷ 2) × 15 = 30.0. The number is not what determines the decision. Axis 2 = 0 records that CCR9 blockade leaves other gut-homing routes intact, so the target proved redundant, and Axis 4 = 0 records that receptor engagement was never linked to mucosal healing; it is that second zero that triggers the hard stop. Applied prospectively, the framework would have called for a non-redundancy experiment in human tissue and an endpoint-linked mucosal readout before Phase 3 commitment, rather than for a larger trial. The retrospective scorecard in Section 6.7 applies the same five steps to every quantitatively scored programme in this review.

The five axes were derived before case scoring by integrating four established components of drug development practice: human genetic target validation, receptor and target-engagement pharmacology, modality-specific PK/PD feasibility, and quality-by-design principles for clinical endpoints and data reliability. The framework is related to the pharmaceutical “5R” approach but is adapted to GPCR-specific considerations, such as subtype selectivity, signaling bias, internalization, and reversible versus prolonged receptor engagement [63,64,65,66,67,68,69,70,71].

A score of 0 denotes evidence that is absent or unacceptable, 1 denotes evidence that is partial, uncertain, or constrained, and 2 denotes evidence that is strong and decision-ready; the axis-specific anchors, and the stage at which each hard stop applies, are set out in Table 1. The weights reflect the expected influence of each axis on a development decision rather than fitted coefficients. Axes 1 and 4 carry the largest weights because a programme that lacks either a causal human anchor or an endpoint-linked readout has no defensible route to a registrational trial. Axis 5 carries a lower weight not because durability matters less, but because it acts chiefly through the hard-stop rule rather than through the weighted total.

The axes interact rather than operate independently. Axis 1 must connect to Axis 4 by defining a responsive population and a mechanism-proximal readout. Axis 2 constrains Axis 3 because subtype selectivity, internalization, and signaling determine the exposure and occupancy profile that is clinically feasible. Axis 3 also constrains Axis 5 because half-life, reversibility, and route of administration determine whether an adverse effect can be managed by dose interruption or requires prolonged monitoring. Axis 5 is stage dependent: evidence reliability is not a biological property of a drug and is applied only after clinical data have been generated.

The scorecard was applied retrospectively to the curated cases included in this review. Established successful programmes scored 80–100, failed programmes scored 30–48, and PAR4 scored 57.5, placing it in the stop-and-rework band. Avacopan scored 80 based on its pre-approval profile but now triggers an Axis 5 hard stop because both safety and evidence reliability have become unacceptable. In a sensitivity analysis using equal 20% weights, PAR4 scores 60.0 and moves to conditional/rework, whereas the classifications of established successes and hard-stop failures are unchanged. This represents retrospective application and sensitivity analysis rather than external validation; prospective, independent validation remains necessary.

**Table 1:** Condensed scoring anchors, weights and stage-specific hard-stop rules for the five-axis framework. Full operational definitions for scores 0, 1 and 2 are provided in Supplementary Table S1. 5A, safety component of Axis 5; 5B, evidence-reliability component of Axis 5; PK/PD, pharmacokinetic/pharmacodynamic.

Axis	Weight	Condensed Score Anchors (0/1/2)	Hard-Stop Use
A1. Human-disease anchoring	25%	No human support/plausible association/causal genetic, anatomic, or human perturbation evidence	A1 = 0: do not nominate without new causal evidence
A2. Receptor pharmacology	15%	Subtype selectivity or redundancy unresolved/partly characterized/selectivity, signaling, and tissue logic defined	No automatic stop; a low score identifies the pharmacology gap
A3. Modality and PK/PD	20%	Clinically infeasible/feasible with major constraints/exposure, route of administration, and reversibility fit the indication	A3 = 0: do not advance clinically
A4. Biomarker to endpoint	25%	No proximal PD or endpoint mismatch/target engagement with uncertain linkage/proximal PD plus a credible endpoint and patient stratification	A4 = 0: stop or redesign the biomarker/endpoint strategy
A5. Durability	15%	Lower of 5A safety and 5B evidence reliability: unacceptable/uncertain or monitorable/acceptable and credible	A5A = 0 or A5B = 0 at registration/post-approval: hold, withdraw, or regenerate evidence

Figure 5 shows the order in which the axes are evaluated, together with three worked tracks illustrating how a programme exits at the axis where its evidence fails; the weights, decision thresholds and hard-stop rules are given in Table 1 and in Supplementary Table S1. It is placed here with the condensed anchors so that readers see the scorecard architecture before the retrospective cases in Section 6.7.

### 6.2. Axis 1—Human-Disease Anchoring

Human genetic evidence or a defined anatomic lesion provides stronger support for causality than receptor expression alone. Across drug development datasets, targets with human genetic support have had higher approval probabilities, with the strongest effects observed for Mendelian associations and protein-altering variants [65,66,67,68,69]. WHIM syndrome, MC4R-pathway obesity, and acquired hypothalamic obesity therefore receive high Axis 1 scores because the eligible population is defined by a causal genotype or lesion.

A strong human anchor also guides patient selection. Genotype- or lesion-based eligibility enriches the trial with patients in whom the mechanism is expected to operate and allows the label to match that population. By contrast, tissue expression or association within a redundant polygenic pathway provides a weaker starting point and requires stronger evidence from the later axes.

### 6.3. Axis 2—Receptor-Subtype Pharmacology

Axis 2 assesses endogenous ligand biology, subtype selectivity, signaling bias, internalization, and tissue distribution. These features determine whether receptor engagement produces the intended response in the relevant tissue.

Subtype selectivity and signaling behavior directly affect the therapeutic window. Unwanted subtype engagement can produce class-specific adverse effects, whereas agonist-induced internalization can result in functional antagonism, as observed with sphingosine-1-phosphate (S1P) receptor modulators. When receptor regulation does not provide a self-limiting effect, the required duration of receptor blockade must instead be controlled through dose and exposure.

### 6.4. Axis 3—Modality and PK/PD Feasibility

Modality is a core determinant of clinical pharmacology. Small molecules, peptides, and biologics differ in exposure duration, reversibility, route of administration, tissue penetration, drug–drug interaction liability, and the degree to which target engagement can be titrated. These properties should be matched to the temporal and safety requirements of the indication.

Acute or intermittent indications favor rapid-onset, reversible exposure, whereas chronic immune-mediated disease often benefits from an agent that can be discontinued and cleared within a clinically useful interval. Long-half-life biologics may be preferable when infrequent dosing and minimal metabolic interactions are more important than rapid reversibility. Section 6.8 provides illustrative comparisons; the calcitonin gene-related peptide (CGRP) receptor illustrates how the same validated target can be addressed with either a monthly monoclonal antibody or an oral small molecule.

### 6.5. Axis 4—Biomarker and Endpoint Readiness

Axis 4 distinguishes target engagement from clinical prediction. A useful PD biomarker should be mechanism-proximal, measurable on the relevant timescale, and linked to the registration endpoint. A biomarker that changes without demonstrating this linkage receives an intermediate or low score rather than being considered proof of translation.

Absolute neutrophil and lymphocyte counts in WHIM syndrome and peripheral lymphocyte counts during S1P receptor modulation directly reflect the mechanism being targeted. In contrast, sputum eosinophils in asthma and histologic fibrosis in steatohepatitis are embedded within more redundant disease biology and did not predict the Phase 3 outcomes reviewed here. The score therefore rewards both mechanistic proximity and endpoint linkage.

### 6.6. Axis 5—Durability: Safety and Evidence Reliability

Axis 5 is a stage-dependent durability gate with two separately recorded components: 5A, pharmacological safety; and 5B, evidence reliability. The axis score is determined by the lower of the two component scores because either unacceptable toxicity or unreliable pivotal evidence can prevent regulatory approval or reverse a favorable post-approval benefit–risk assessment.

Safety and evidence reliability are considered separately because they require different responses. A safety problem may sometimes be mitigated through dose adjustment, monitoring, patient selection, or the use of a more reversible modality. An evidence reliability failure cannot be corrected by dose adjustment; instead, the pivotal analysis must be independently verified or repeated. Evidence reliability is therefore a clinical development governance checkpoint rather than a biological property used to rank preclinical targets. Avacopan is included only as a post-approval stress test of this gate.

### 6.7. Retrospective Scorecard and Evidence Matrix

Table 2 reports the source-based scores used for the retrospective application. The values represent transparent judgments based on the evidence summarized in the Results and, for selected programmes, in Table 3 and Table 4; they are not estimates of approval probability.

### 6.8. Modality and PK/PD Feasibility: Worked Comparisons

Axis 3 is assessed using explicit, comparable parameters: exposure duration, PD reversibility after discontinuation, route of administration, drug–drug interaction liability, target engagement control, and selectivity. These dimensions should not be collapsed into a single occupancy metric. Four worked comparisons illustrate how the same target biology can require different pharmaceutical solutions.

Reversible oral small molecule with a controllable PD response. Etrasimod is a once-daily oral S1P modulator with an elimination half-life of approximately 30–36 h, dose-proportional exposure, and steady state achieved by day 7. Peripheral lymphocyte counts decrease during treatment and generally return to the normal range within approximately 7 days after discontinuation [72]. This reversible PD profile permits withdrawal-based management of infection, cardiac conduction, or hepatic concerns, although CYP-mediated metabolism creates drug interaction and labeling requirements.

Twice-daily oral small molecule with metabolic and hepatic constraints. Avacopan is administered at 30 mg twice daily and is affected by CYP3A4-mediated drug interactions. Its exposure profile therefore intersects with liver monitoring and concomitant medication management, although the current regulatory reassessment cannot be attributed to PK alone.

Peptide requiring parenteral delivery. Setmelanotide is administered by once-daily subcutaneous injection [73]. The route of administration is a practical development constraint, and melanocortin family cross-reactivity contributes to characteristic exposure-related effects such as hyperpigmentation. This example links Axis 2 selectivity with Axis 3 route of administration and exposure.

Same target, different modality profiles. Erenumab is a monoclonal antibody against the CGRP receptor with a terminal half-life measured in weeks, enabling monthly subcutaneous dosing and minimal CYP-mediated interactions but producing a slow onset and offset. Gepants such as rimegepant target the same receptor as oral small molecules with faster and more reversible exposure [47,48,74]. The choice between these modalities depends on the clinical use case rather than on target validity alone.

Axis 3 should therefore be scored using prespecified PK/PD and delivery parameters rather than a broad modality label. Exposure duration and reversibility describe temporal characteristics; target engagement control describes titratability; and receptor subtype or signaling pathway selectivity describes pharmacological scope.

### 6.9. Use of Generative AI-Assisted Tools

During manuscript revision, the authors used Claude (Anthropic; Claude Opus 5, Max subscription tier) and ChatGPT (OpenAI; GPT-5.6, Plus subscription tier), between August and September 2026, for language refinement, structural editing, internal consistency checks, and bibliographic metadata cross-checking. These tools were not used to generate or alter primary experimental data and did not independently determine scientific interpretations or conclusions. All AI-assisted output was reviewed by the authors and checked against the cited primary or regulatory sources before inclusion.

## 7. Conclusions

GPCR translation depends on a causal human anchor, receptor pharmacology that can be implemented through a feasible therapeutic modality and exposure profile, a mechanism-proximal biomarker linked to the clinical endpoint, and durable safety supported by reliable evidence. The revised framework converts these considerations into an explicit scoring system, interaction checks, and a stage-specific hard-stop process.

The framework should not be interpreted as a validated probability model. Its value is procedural: evidence gaps are identified before the next development decision, critical weaknesses cannot be averaged away, and each low score points to a specific corrective action. Applied prospectively, the scorecard can support more transparent prioritization while emphasizing that de-risking continues through regulatory approval and post-approval surveillance.

## Figures and Tables

**Figure 1:**
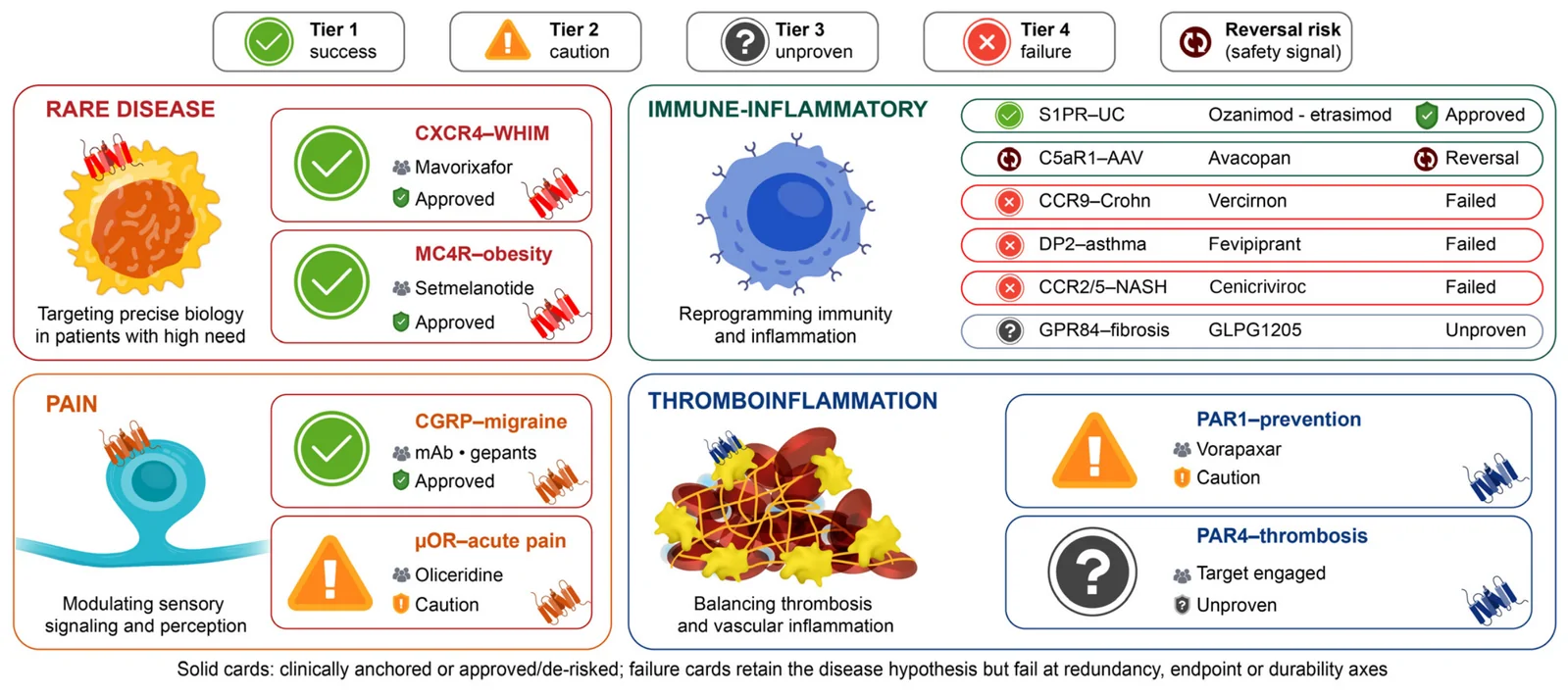
Clinically anchored GPCR landscape across four therapeutic domains. Programmes are grouped by disease domain and representative modality. The icon key at the top of the figure denotes the evidence tier assigned to each programme: green circle with a check mark, Tier 1 (success); amber triangle with an exclamation mark, Tier 2 (caution); grey circle with a question mark, Tier 3 (unproven); red circle with a cross, Tier 4 (failure); dark-red circular arrows, reversal risk (post-approval safety signal). The same icons are repeated in reduced form within each programme card to show its current status. Panel and card border colours identify the four disease domains and carry no evidential meaning. The landscape is descriptive: of the thirteen programmes reviewed, eleven are quantitatively scored in the retrospective scorecard (Section 6.7), whereas oliceridine is shown as a qualitative comparator and avexitide is discussed in the text but is not depicted. AAV, ANCA-associated vasculitis; mAb, monoclonal antibody; NASH, nonalcoholic steatohepatitis; S1PR, sphingosine-1-phosphate receptor; UC, ulcerative colitis; WHIM, warts, hypogammaglobulinaemia, infections and myelokathexis syndrome; μOR, μ-opioid receptor.

**Figure 2:**
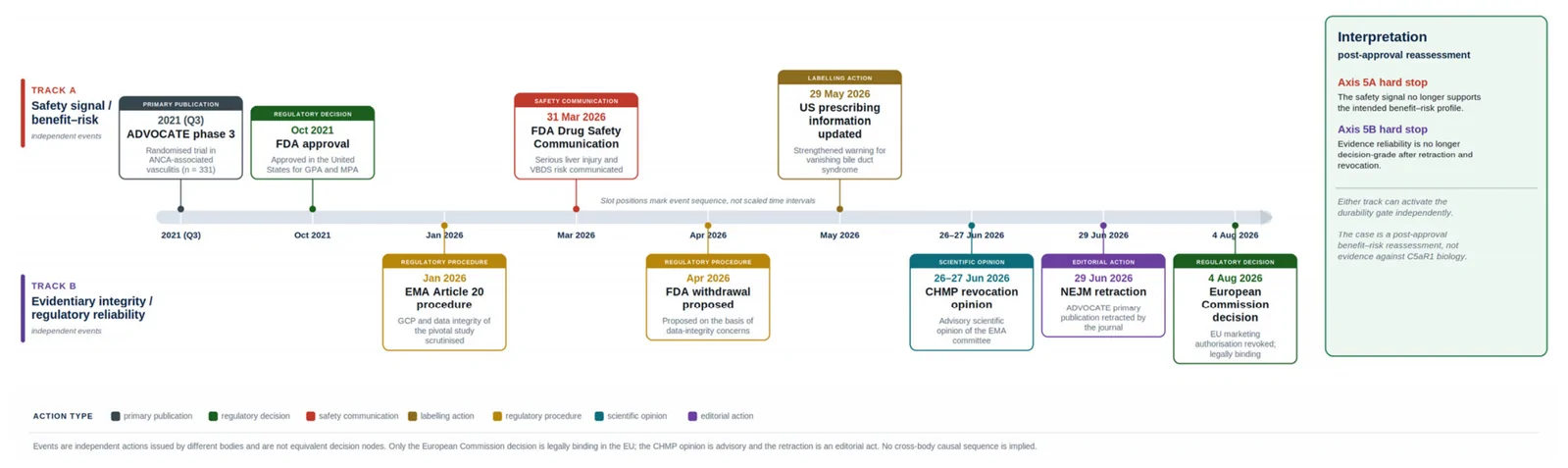
C5aR1/avacopan parallel post-approval stress-test tracks. Safety-signal and evidentiary-integrity events are separated because they represent different de-risking failures. Calendar alignment indicates timing only: FDA, EMA/CHMP, European Commission, and NEJM actions are independent events issued by different bodies and are not equivalent nodes; no causal ordering across tracks is implied. The case is framed as a post-approval benefit–risk reassessment rather than as evidence disproving complement/C5aR1 biology. Colours in the action-type key identify the kind of action taken (primary publication, regulatory decision, safety communication, labelling action, regulatory procedure, scientific opinion, editorial action) and do not encode severity; the two track labels (red, safety signal and benefit–risk; purple, evidentiary integrity and regulatory reliability) identify the track only. Slot positions mark event sequence, not scaled time intervals. CHMP, Committee for Medicinal Products for Human Use; EMA, European Medicines Agency; FDA, U.S. Food and Drug Administration; GCP, Good Clinical Practice; GPA, granulomatosis with polyangiitis; MPA, microscopic polyangiitis; NEJM, New England Journal of Medicine; VBDS, vanishing bile duct syndrome.

**Figure 3:**
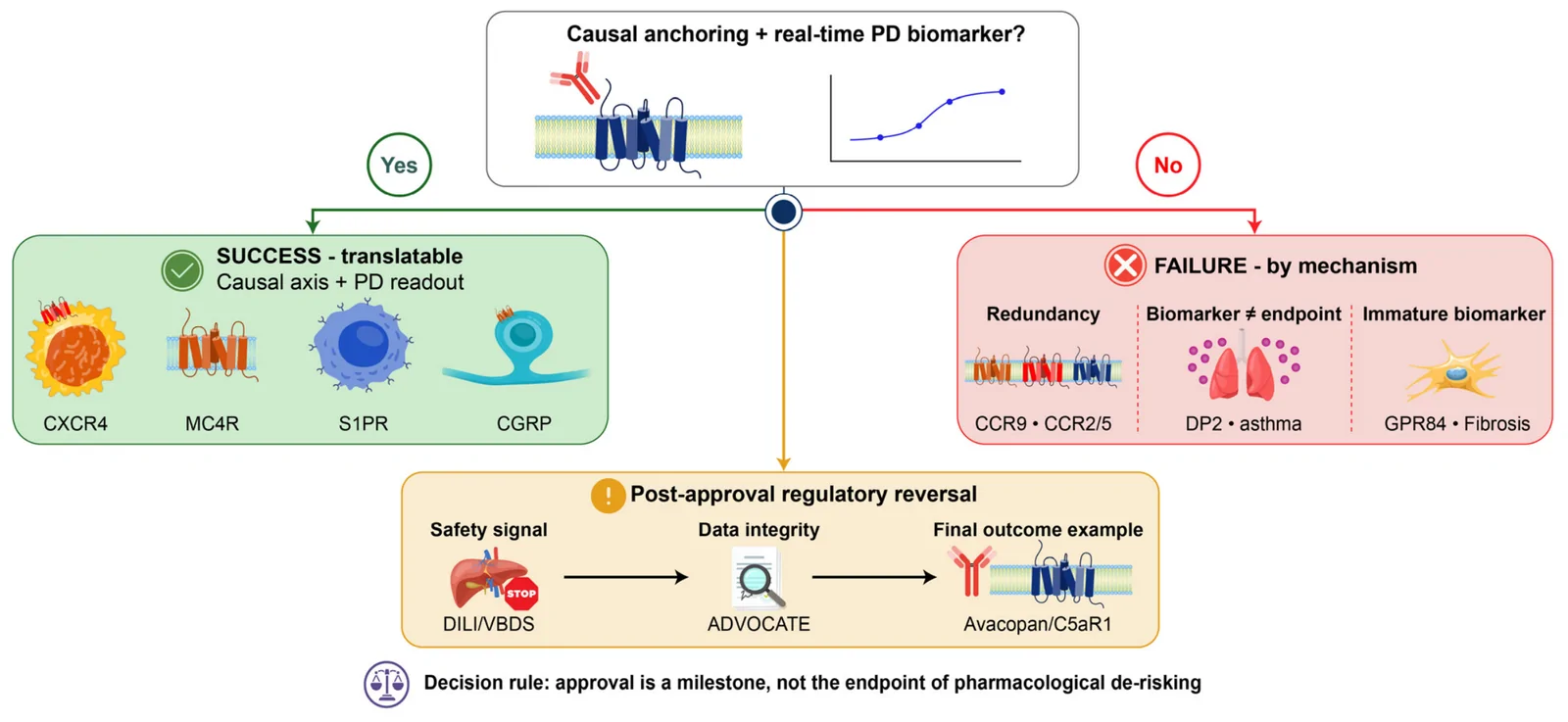
Decision rule for clinically anchored GPCR programmes. Programmes that combine a causal human-disease anchor with a mechanism-proximal pharmacodynamic readout translated successfully (CXCR4, MC4R, S1PR, CGRP), whereas programmes lacking one of these elements failed, and the mechanism of failure differs: receptor redundancy (CCR9, CCR2/CCR5), a biomarker that is not the endpoint (DP2) and an immature clinical biomarker (GPR84). A third branch shows post-approval regulatory reversal, in which a safety signal and a loss of confidence in the pivotal evidence acted together (avacopan/C5aR1). Approval is therefore treated as a milestone in de-risking rather than its endpoint. Green shading and the green circled check mark indicate programmes in which both a causal human-disease anchor and a mechanism-proximal pharmacodynamic readout are present; red shading and the red circled cross indicate failure, subdivided by mechanism; amber shading and the amber circled exclamation mark indicate post-approval regulatory reversal. The green and red branch labels (Yes, No) refer to the question posed in the uppermost box. Arrows show the direction of the decision path and do not imply a temporal sequence. Dashed vertical rules separate the three failure mechanisms. DILI, drug-induced liver injury; PD, pharmacodynamic; VBDS, vanishing bile duct syndrome.

**Figure 4:**
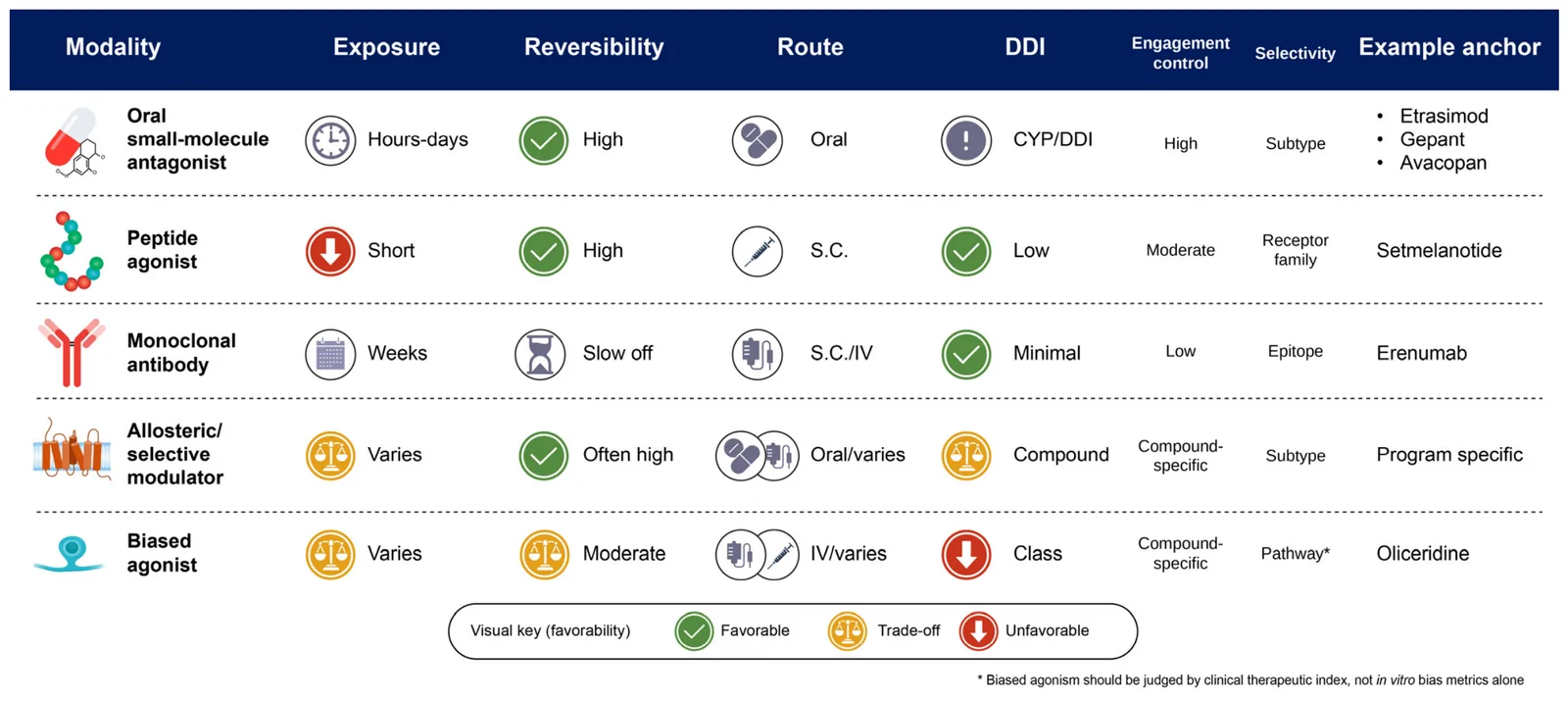
Modality–PK/PD map for GPCR therapeutics. Exposure duration and PD reversibility describe temporal characteristics; target engagement control describes dose or exposure titratability; and selectivity describes receptor subtype, epitope, or signaling pathway scope. These dimensions are presented separately to avoid treating pathway bias or subtype selectivity as measures of target engagement. Within each column, symbols indicate favourability: green circled check mark, favourable; amber circled balance, trade-off; red circled downward arrow, unfavourable, as stated in the visual key at the foot of the figure. The clock, calendar, hourglass, tablet, syringe and infusion-bag icons are pictograms for the adjacent text and carry no additional meaning. Row shading distinguishes modalities only. DDI, drug–drug interaction; IV, intravenous; s.c., subcutaneous.

**Figure 5:**
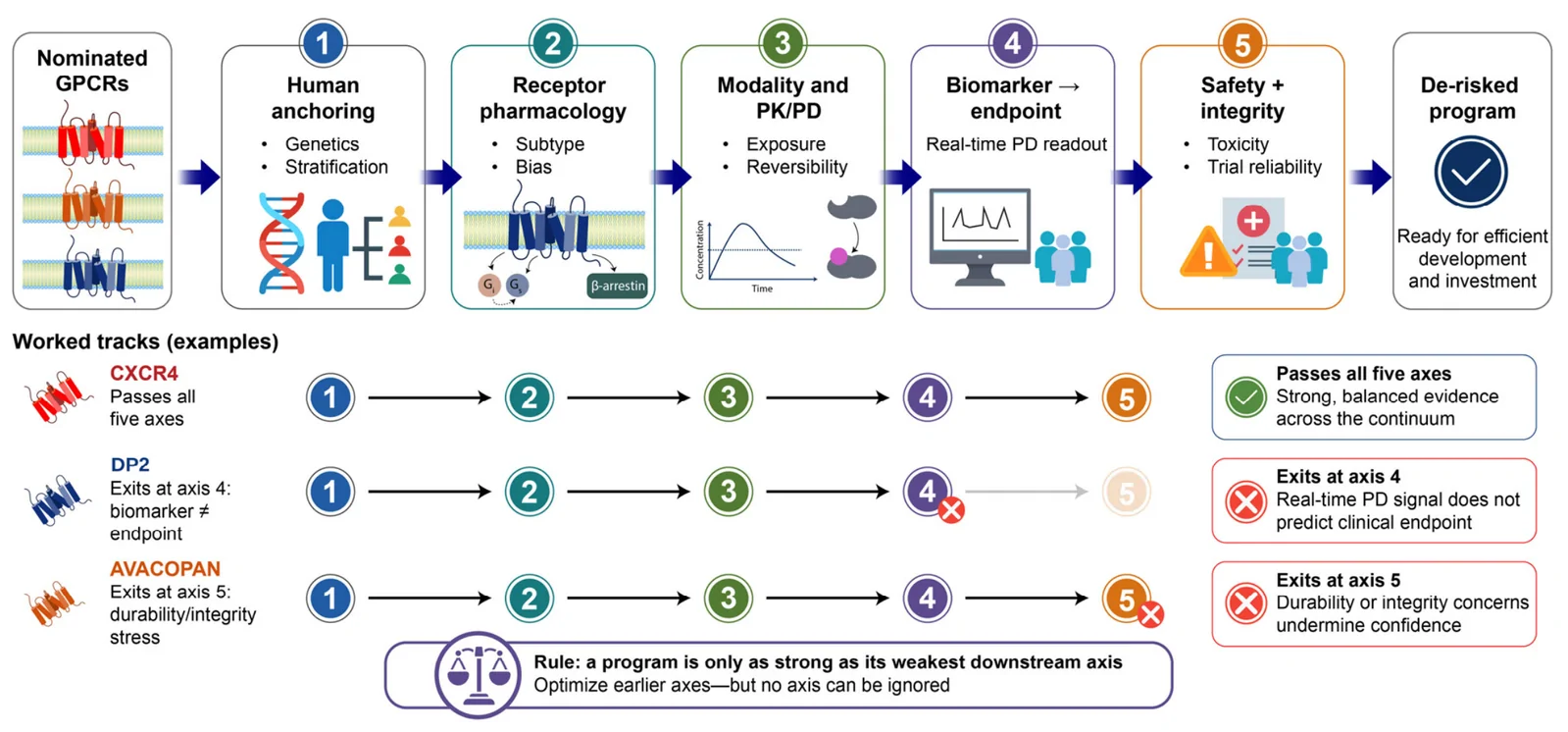
Operational five-axis GPCR translational scorecard. Each axis is scored from 0 to 2 and weighted to yield a nominal 100-point total when all axes are assessable. Axis 5 records safety (5A) and evidence reliability (5B) separately and uses the lower component score once both are assessable. Scores of ≥75, 60–74, and <60 indicate advance, conditional/rework, and stop/rework, respectively, but any hard stop overrides the total score. The retrospective application shown here is illustrative and is not an externally validated prediction model. In the upper panel, arrows indicate the order in which the axes are evaluated. In the worked tracks, a solid arrow indicates progression to the next axis, whereas a faded arrow leading to a faded node indicates an axis that is not reached because the programme exits at the preceding axis, marked by a red circled cross. The green circled check mark in the worked-track key and the dark-blue circled check mark in the De-risked program panel denote the same outcome—all five axes satisfied—and differ only in position. Axis colours are used for identification only and do not encode rank or weight.

**Table 2:** Retrospective application of the operational five-axis GPCR scorecard.

Programme/Modality	A1	A2	A3	A4	A5A/A5B → A5	Score/100	Framework Decision; Observed Status
CXCR4–WHIM (oral small molecule)	2	2	2	2	1/2 → 1	92.5	Advance; approved
MC4R–obesity (peptide, s.c.)	2	2	1	2	1/2 → 1	82.5	Advance; approved
S1PR–UC (oral small molecule)	1	2	2	2	1/2 → 1	80.0	Advance; approved
CGRP–migraine (mAb and oral small molecule)	2	2	2	2	2/2 → 2	100	Advance; approved across modalities
C5aR1–AAV (current) (oral small molecule)	2	2	2	1	0/0 → 0	72.5 *	Hard stop; EU approval revoked; FDA withdrawal proposed
PAR1–thrombosis (oral small molecule)	2	2	1	2	1/2 → 1	82.5	Advance with major safety constraints; approved
PAR4–thrombosis (oral small molecule)	1	2	1	1	1/2 → 1	57.5	Stop/rework; target engaged, outcome unproven
CCR9–Crohn disease (oral small molecule)	1	0	1	0	1/2 → 1	30.0 *	Hard stop; failed Phase 3
DP2–asthma (oral small molecule)	1	1	2	0	1/2 → 1	47.5 *	Hard stop; failed Phase 3
CCR2/CCR5–NASH (oral small molecule)	1	1	1	0	1/2 → 1	37.5 *	Hard stop; failed Phase 3
GPR84–fibrosis (oral small molecule)	0	1	1	0	1/NA → 1	25.0 *	Hard stop; early/limited clinical evidence

* Hard-stop rule overrides the numerical total. A5A, safety; A5B, evidence reliability; NA, not yet assessable. For a wholly NA axis, that axis is excluded from the numerator and assessable-weight denominator and the total is normalized to the remaining assessable weight. If one Axis 5 component is genuinely NA, the provisional Axis 5 score is based on the assessable component and reported as x/NA; once both components are assessable, Axis 5 is the lower component score. The modality of each programme is given under the programme name. Weighted total = Σ (axis score ÷ 2) × axis weight, with weights of 25% (A1), 15% (A2), 20% (A3), 25% (A4), and 15% (A5). A1, human-disease anchoring; A2, receptor-subtype pharmacology; A3, modality and PK/PD feasibility; A4, biomarker-to-endpoint readiness; A5, durability. AAV, ANCA-associated vasculitis; ANCA, anti-neutrophil cytoplasmic antibody; mAb, monoclonal antibody; NASH, nonalcoholic steatohepatitis; PK/PD, pharmacokinetic/pharmacodynamic; s.c., subcutaneous; UC, ulcerative colitis; WHIM, warts, hypogammaglobulinaemia, infections and myelokathexis syndrome.

**Table 3:** Clinically anchored GPCR programmes: score-relevant evidence and development implication.

Programme/Modality	Key Clinical or Regulatory Evidence	Principal Limitation	Score/Implication
CXCR4–WHIM Mavorixafor; oral small molecule	4WHIM Phase 3: increased TAT-ANC/ALC and reduced infection measures [9,10,11,12,13]. FDA approval, April 2024.	Small orphan population; warts unchanged; long-term outcomes remain limited.	92.5/100. Advance; continue long-term follow-up.
MC4R–obesity Setmelanotide; peptide, s.c.	Clinical benefit in genetically defined obesity; indication extended to acquired hypothalamic obesity in 2026 [14,15,16,17].	Daily injection; receptor-family effects and neuropsychiatric monitoring.	82.5/100. Advance in genetically or anatomically defined populations.
S1PR–UC Ozanimod/etrasimod; oral small molecules	Approved for ulcerative colitis; lymphocyte reduction provides mechanism-proximal PD [20,21,22,23,24,25,26,27,28].	Modest remission rates; cardiac, ocular, hepatic, and infection monitoring.	80.0/100. Advance with class-specific safety monitoring.
C5aR1–AAV Avacopan; oral small molecule	ADVOCATE supported approval [29]; post-approval serious DILI/VBDS and loss of confidence in pivotal evidence led to an FDA withdrawal proposal and EU revocation on 4 August 2026 [30,31,32].	Distinct safety and evidentiary-integrity liabilities.	72.5/100 with Axis 5 hard stop. Regenerate efficacy evidence and reassess benefit–risk.

**Table 4:** Failed or unproven GPCR programmes: score-localized evidence gaps and requirements for reconsideration.

Programme/Modality	Outcome and Score-Localized Gap	Score/Hard Stop	Evidence Required Before Reconsideration
CCR9–Crohn disease Vercirnon; oral antagonist	Positive Phase 2 was not replicated in Phase 3; receptor redundancy and absence of tissue-level occupancy/trafficking linkage remained unresolved [33,34,35,36,37].	30.0/100; A4 hard stop.	Demonstrate mucosal target engagement, nonredundant pathogenic-cell trafficking control, and prospective enrichment.
DP2–asthma Fevipiprant; oral antagonist	Sputum eosinophils changed, but Phase 3 exacerbation endpoints were not improved; biomarker-to-endpoint linkage was not established [38,39,40].	47.5/100; A4 hard stop.	Define a responsive phenotype and prospectively show that the PD biomarker predicts exacerbation benefit.
CCR2/CCR5–NASH Cenicriviroc; oral dual antagonist	Phase 2b antifibrotic signal did not translate to Phase 3 efficacy; histology remained an insufficient outcome bridge [41,42,43].	37.5/100; A4 hard stop.	Establish liver target engagement/exposure-response and a surrogate or endpoint with demonstrated outcome relevance.
GPR84–fibrosis GLPG1205; oral small molecule	Early/limited clinical evidence; no endpoint-linked PD package sufficient for late-stage commitment [44].	25.0/100; hard stop; A5 = 1/NA provisional.	Generate clinical target-engagement and endpoint-linkage evidence before advancement.
PAR4–thrombosis BMS-986120-class; oral small molecule	Human PD target engagement demonstrated, but no outcome trial establishes antithrombotic efficacy with an acceptable bleeding window [61].	57.5/100; stop/rework.	Establish exposure–response, efficacy–bleeding separation, and outcome-relevant evidence before an outcome trial.

## Data Availability

No new data were created or analyzed in this study. Data sharing is not applicable to this article.

## Supplementary Materials

The following supporting information can be downloaded at: https://www.mdpi.com/article/10.3390/ph19101510/s1, Table S1: Expanded operational definitions for the five-axis GPCR translational scorecard.

## References

[1] Hauser A.S., Attwood M.M., Rask-Andersen M., Schiöth H.B., Gloriam D.E. (2017). Trends in GPCR drug discovery: New agents, targets and indications. Nat. Rev. Drug Discov..

[2] Lorente J.S., Sokolov A.V., Ferguson G., Schiöth H.B., Hauser A.S., Gloriam D.E. (2025). GPCR drug discovery: New agents, targets and indications. Nat. Rev. Drug Discov..

[3] Congreve M., de Graaf C., Swain N.A., Tate C.G. (2020). Impact of GPCR structures on drug discovery. Cell.

[4] Sriram K., Insel P.A.G. (2018). G Protein-coupled receptors as targets for approved drugs: How many targets and how many drugs?. Mol. Pharmacol..

[5] Zhang M., Chen T., Lu X., Lan X., Chen Z., Lu S. (2024). G protein-coupled receptors (GPCRs): Advances in structures, mechanisms and drug discovery. Signal Transduct. Target. Ther..

[6] McDermott D.H., Murphy P.M. (2019). WHIM syndrome: Immunopathogenesis, treatment and cure strategies. Immunol. Rev..

[7] Heusinkveld L.E., Majumdar S., Gao J.-L., McDermott D.H., Murphy P.M. (2019). WHIM syndrome: From pathogenesis towards personalized medicine and cure. J. Clin. Immunol..

[8] Balabanian K., Lagane B., Pablos J.L., Laurent L., Planchenault T., Verola O., Lebbe C., Kerob D., Dupuy A., Hermine O. (2005). WHIM syndromes with different genetic anomalies are accounted for by impaired CXCR4 desensitization to CXCL12. Blood.

[9] X4 Pharmaceuticals (2024). Xolremdi (Mavorixafor) Prescribing Information. [FDA Approval 26 April 2024].

[10] X4 Pharmaceuticals (2024). FDA Approval of XOLREMDI (Mavorixafor) for WHIM Syndrome [Press Release], 29 April 2024.

[11] Badolato R., Alsina L., Azar A., Bertrand Y., Bolyard A.A., Dale D., Deyà-Martínez À., Dickerson K.E., Ezra N., Hasle H. (2024). A phase 3 randomized trial of mavorixafor, a CXCR4 antagonist, for WHIM syndrome. Blood.

[12] Dale D.C., Firkin F., Bolyard A.A., Kelley M., Makaryan V., Gorelick K.J., Ebrahim T., Garg V., Tang W., Jiang H. (2020). Results of a phase 2 trial of an oral CXCR4 antagonist, mavorixafor, for treatment of WHIM syndrome. Blood.

[13] McDermott D.H., Liu Q., Velez D., Lopez L., Anaya-O’Brien S., Ulrick J., Kwatemaa N., Starling J., Fleisher T.A., Priel D.A.L. (2014). A phase 1 clinical trial of long-term, low-dose treatment of WHIM syndrome with the CXCR4 antagonist plerixafor. Blood.

[14] Clément K., van den Akker E., Argente J., Bahm A., Chung W.K., Connors H., De Waele K., Farooqi I.S., Gonneau-Lejeune J., Gordon G. (2020). Efficacy and safety of setmelanotide, an MC4R agonist, in individuals with severe obesity due to LEPR or POMC deficiency: Single-arm, open-label, multicentre, phase 3 trials. Lancet Diabetes Endocrinol..

[15] US Food and Drug Administration Supplemental New Drug Application Approval Letter for IMCIVREE (Setmelanotide), NDA 213793/S-009. https://www.fda.gov/drugs.

[16] Haqq A.M., Chung W.K., Dollfus H., Haws R.M., Martos-Moreno G.Á., Poitou C., Yanovski J.A., Mittleman R.S., Yuan G., Forsythe E. (2022). Efficacy and safety of setmelanotide, a melanocortin-4 receptor agonist, in patients with Bardet-Biedl syndrome and Alström syndrome: A multicentre, randomised, double-blind, placebo-controlled, phase 3 trial with an open-label period. Lancet Diabetes Endocrinol..

[17] Rhythm Pharmaceuticals, Inc. (2026). IMCIVREE (Setmelanotide) Injection, for Subcutaneous Use, Prescribing Information.

[18] Salehi M., Gastaldelli A., D’Alessio D.A. (2014). Blockade of glucagon-like peptide 1 receptor corrects postprandial hypoglycemia after gastric bypass. Gastroenterology.

[19] Craig C.M., Lawler H.M., Lee C.J.E., Tan M., Davis D.B., Tong J., Glodowski M., Rogowitz E., Karaman R., McLaughlin T.L. (2021). PREVENT: A randomized, placebo-controlled crossover trial of avexitide for treatment of postbariatric hypoglycemia. J. Clin. Endocrinol. Metab..

[20] Sandborn W.J., Feagan B.G., D’haens G., Wolf D.C., Jovanovic I., Hanauer S.B., Ghosh S., Petersen A., Hua S.Y., Lee J.H. (2021). Ozanimod as induction and maintenance therapy for ulcerative colitis. N. Engl. J. Med..

[21] Sandborn W.J., Vermeire S., Peyrin-Biroulet L., Dubinsky M.C., Panes J., Yarur A., Ritter T., Baert F., Schreiber S., Sloan S. (2023). Etrasimod as induction and maintenance therapy for ulcerative colitis (ELEVATE): Two randomised, double-blind, placebo-controlled, phase 3 studies. Lancet.

[22] Sandborn W.J., Feagan B.G., Wolf D.C., D’Haens G., Vermeire S., Hanauer S.B., Ghosh S., Smith H., Cravets M., Frohna P.A. (2016). Ozanimod induction and maintenance treatment for ulcerative colitis. N. Engl. J. Med..

[23] Pfizer (2023). Velsipity (Etrasimod) Prescribing Information. [FDA Approval October 2023].

[24] Bristol-Myers Squibb Company (2021). ZEPOSIA (Ozanimod) Capsules, Prescribing Information.

[25] Matloubian M., Lo C.G., Cinamon G., Lesneski M.J., Xu Y., Brinkmann V., Allende M.L., Proia R.L., Cyster J.G. (2004). Lymphocyte egress from thymus and peripheral lymphoid organs is dependent on S1P receptor 1. Nature.

[26] Chun J., Kihara Y., Jonnalagadda D., Blaho V.A. (2019). Fingolimod: Lessons learned and new opportunities for treating multiple sclerosis and other disorders. Annu. Rev. Pharmacol. Toxicol..

[27] Al-Shamma H., Lehmann-Bruinsma K., Carroll C., Solomon M., Komori H.K., Peyrin-Biroulet L., Adams J. (2019). The selective sphingosine 1-phosphate receptor modulator etrasimod regulates lymphocyte trafficking and alleviates experimental colitis. J. Pharmacol. Exp. Ther..

[28] Paik J. (2022). Ozanimod: A review in ulcerative colitis. Drugs.

[29] Jayne D.R.W., Merkel P.A., Schall T.J., Bekker P., ADVOCATE Study Group (2021). Avacopan for the treatment of ANCA-associated vasculitis. N. Engl. J. Med..

[30] US Food and Drug Administration FDA Identifies Cases of Serious Liver Injury in Patients Taking Tavneos (Avacopan) for Severe Active Anti-Neutrophil Cytoplasmic Autoantibody (ANCA)-Associated Vasculitis; Drug Safety Communication, 31 March 2026. https://www.fda.gov/drugs/drug-safety-communications/fda-identifies-cases-serious-liver-injury-patients-taking-tavneos-avacopan-severe-active-anti.

[31] Rubin E.J. (2026). Retraction: Jayne DRW et al. Avacopan for the Treatment of ANCA-Associated Vasculitis, N Engl J Med 2021;384:599-609. N. Engl. J. Med..

[32] European Medicines Agency EMA Recommends Revoking Marketing Authorisation for Tavneos (Avacopan); CHMP Opinion, Article 20 Referral, 26 June 2026; the European Commission Issued its Final Legally Binding Revocation Decision on 4 August 2026. https://www.ema.europa.eu/en/news/ema-recommends-revoking-marketing-authorisation-tavneos.

[33] Feagan B.G., Sandborn W.J., D’haens G., Lee S.D., Allez M., Fedorak R.N., Seidler U., Vermeire S., Lawrance I.C., Maroney A.C. (2015). Randomised clinical trial: Vercirnon, an oral CCR9 antagonist, vs. placebo as induction therapy in active Crohn’s disease. Aliment. Pharmacol. Ther..

[34] Keshav S., Vaňásek T., Niv Y., Petryka R., Howaldt S., Bafutto M., Rácz I., Hetzel D., Nielsen O.H., Vermeire S. (2013). PROTECT-1 study group. A randomized controlled trial of the efficacy and safety of the oral CCR9 antagonist vercirnon (CCX282-B) in patients with Crohn’s disease (PROTECT-1). PLoS ONE.

[35] Feagan B.G., Rutgeerts P., Sands B.E., Hanauer S., Colombel J.F., Sandborn W.J., Van Assche G., Axler J., Kim H.J., Danese S. (2013). Vedolizumab as induction and maintenance therapy for ulcerative colitis. N. Engl. J. Med..

[36] Sandborn W.J., Feagan B.G., Rutgeerts P., Hanauer S., Colombel J.F., Sands B.E., Lukas M., Fedorak R.N., Lee S., Bressler B. (2013). Vedolizumab as induction and maintenance therapy for Crohn’s disease. N. Engl. J. Med..

[37] Wyant T., Fedyk E., Abhyankar B. (2016). An overview of the mechanism of action of the monoclonal antibody vedolizumab. J. Crohns Colitis.

[38] Brightling C.E., Gaga M., Inoue H., Li J., Maspero J., Wenzel S., Maitra S., Lawrence D., Brockhaus F., Lehmann T. (2021). Effectiveness of fevipiprant in reducing exacerbations in patients with severe asthma (LUSTER-1 and LUSTER-2): Two phase 3 randomised controlled trials. Lancet Respir. Med..

[39] Castro M., Kerwin E., Miller D., Pedinoff A., Sher L., Cardenas P., Knorr B., Lawrence D., Ossa D., Wang W. (2021). Efficacy and safety of fevipiprant in patients with uncontrolled asthma: Two replicate, phase 3, randomised, double-blind, placebo-controlled trials (ZEAL-1 and ZEAL-2). EClinicalmedicine.

[40] Gonem S., Berair R., Singapuri A., Hartley R., Laurencin M.F.M., Bacher G., Holzhauer B., Bourne M., Mistry V., Pavord I.D. (2016). Fevipiprant, a prostaglandin D2 receptor 2 antagonist, in patients with persistent eosinophilic asthma: A single-centre, randomised, double-blind, parallel-group, placebo-controlled trial. Lancet Respir. Med..

[41] Ratziu V., Sanyal A., Harrison S.A., Wong V.W.S., Francque S., Goodman Z., Aithal G.P., Kowdley K.V., Seyedkazemi S., Fischer L. (2020). Cenicriviroc treatment for adults with nonalcoholic steatohepatitis and fibrosis: Final analysis of the Phase 2b CENTAUR study. Hepatology.

[42] Anstee Q.M., Neuschwander-Tetri B.A., Wai-Sun Wong V.W.-S., Abdelmalek M.F., Rodriguez-Araujo G., Landgren H., Park G.S., Bedossa P., Alkhouri N., Tacke F. (2024). Cenicriviroc lacked efficacy to treat liver fibrosis in nonalcoholic steatohepatitis: Aurora Phase III randomized study. Clin. Gastroenterol. Hepatol..

[43] Anstee Q.M., Neuschwander-Tetri B.A., Wong V.W.-S., Abdelmalek M.F., Younossi Z.M., Yuan J., Pecoraro M.L., Seyedkazemi S., Fischer L., Bedossa P. (2020). Cenicriviroc for the treatment of liver fibrosis in adults with nonalcoholic steatohepatitis: AURORA Phase 3 study design. Contemp. Clin. Trials.

[44] Strambu I.R., Seemayer C.A., Fagard L.M.A., Ford P.A., Van der Aa T.A.K., de Haas-Amatsaleh A.A., Modgill V., Santermans E., Sondag E.N., Helmer E.G. (2023). GLPG1205 for idiopathic pulmonary fibrosis: A phase 2 randomised placebo-controlled trial. Eur. Respir. J..

[45] Wang J., Wu X., Simonavicius N., Tian H., Ling L. (2006). Medium-chain fatty acids as ligands for orphan G protein-coupled receptor GPR84. J. Biol. Chem..

[46] Marsango S., Barki N., Jenkins L., Tobin A.B., Milligan G. (2022). Therapeutic validation of an orphan G protein-coupled receptor: The case of GPR84. Br. J. Pharmacol..

[47] Goadsby P.J., Reuter U., Hallström Y., Broessner G., Bonner J.H., Zhang F., Sapra S., Picard H., Mikol D.D., Lenz R.A. (2017). A controlled trial of erenumab for episodic migraine. N. Engl. J. Med..

[48] Croop R., Lipton R.B., Kudrow D., Stock D.A., Kamen L., Conway C.M., Stock E.G., Coric V., Goadsby P.J. (2021). Oral rimegepant for preventive treatment of migraine: A phase 2/3, randomised, double-blind, placebo-controlled trial. Lancet.

[49] Lipton R.B., Croop R., Stock E.G., Stock D.A., Morris B.A., Frost M., Dubowchik G.M., Conway C.M., Coric V., Goadsby P.J. (2019). Rimegepant, an oral calcitonin gene-related peptide receptor antagonist, for migraine. N. Engl. J. Med..

[50] Edvinsson L., Haanes K.A., Warfvinge K., Krause D.N. (2018). CGRP as the target of new migraine therapies—Successful translation from bench to clinic. Nat. Rev. Neurol..

[51] Amgen (2018). Aimovig (Erenumab) Prescribing Information.

[52] Biohaven Pharmaceuticals (2021). NURTEC ODT (Rimegepant) Orally Disintegrating Tablets, Prescribing Information.

[53] Viscusi E.R., Skobieranda F., Soergel D.G., Cook E., Burt D.A., Singla N. (2019). APOLLO-1: A randomized placebo- and active-controlled phase III study of oliceridine (TRV130), a G protein-biased ligand at the mu-opioid receptor, for moderate-to-severe acute pain following bunionectomy. J. Pain Res..

[54] Bergese S.D., Brzezinski M., Hammer G.B., Beard T.L., Pan P.H., Mace S.E., Berkowitz R.D., Cochrane K., Wase L., Minkowitz H.S. (2019). Athena: A phase 3, open-label study of oliceridine in moderate-to-severe acute pain requiring parenteral opioid therapy. J. Pain Res..

[55] Trevena, Inc. (2020). OLINVYK (Oliceridine) Injection, Prescribing Information.

[56] DeWire S.M., Yamashita D.S., Rominger D.H., Liu G., Cowan C.L., Graczyk T.M., Chen X.T., Pitis P.M., Gotchev D., Yuan C. (2013). A G protein-biased ligand at the μ-opioid receptor is potently analgesic with reduced gastrointestinal and respiratory dysfunction compared with morphine. J. Pharmacol. Exp. Ther..

[57] Gillis A., Kliewer A., Kelly E., Henderson G., Christie M.J., Schulz S., Canals M. (2020). Critical assessment of G protein-biased agonism at the μ-opioid receptor. Trends Pharmacol. Sci..

[58] Morrow D.A., Braunwald E., Bonaca M.P., Ameriso S.F., Dalby A.J., Fish M.P., Fox K.A.A., Lipka L.J., Liu X., Nicolau J.C. (2012). Vorapaxar in the secondary prevention of atherothrombotic events. N. Engl. J. Med..

[59] (2014). ZONTIVITY (Vorapaxar) Tablets, Prescribing Information (Boxed Warning for Bleeding).

[60] Scirica B.M., Bonaca M.P., Braunwald E., De Ferrari G.M., Isaza D., Lewis B.S., Mehrhof F., Merlini P.A., Murphy S.A., Sabatine M.S. (2012). Vorapaxar for secondary prevention of thrombotic events for patients with previous myocardial infarction: A prespecified subgroup analysis of the TRA 2°P-TIMI 50 trial. Lancet.

[61] Wilson S.J., Ismat F.A., Wang Z., Cerra M., Narayan H., Raftis J., Gray T.J., Connell S., Garonzik S., Ma X. (2018). PAR4 (protease-activated receptor 4) antagonism with BMS-986120 inhibits human ex vivo thrombus formation. Arterioscler. Thromb. Vasc. Biol..

[62] Wong P.C., Seiffert D., Bird J.E., Watson C.A., Bostwick J.S., Giancarli M., Allegretto N., Hua J., Harden D., Guay J. (2017). Blockade of protease-activated receptor-4 (PAR4) provides robust antithrombotic activity with low bleeding. Sci. Transl. Med..

[63] International Council for Harmonisation (2021). ICH E8: General Considerations for Clinical Studies, Step 4 Guideline.

[64] International Council for Harmonisation of Technical Requirements for Pharmaceuticals for Human Use (2025). ICH Harmonised Guideline E6(R3): Good Clinical Practice—Principles and Annex 1, Step 4 Version.

[65] Trajanoska K., Bhérer C., Taliun D., Zhou S., Richards J.B., Mooser V. (2023). From target discovery to clinical drug development with human genetics. Nature.

[66] Plenge R.M., Scolnick E.M., Altshuler D. (2013). Validating therapeutic targets through human genetics. Nat. Rev. Drug Discov..

[67] Nelson M.R., Tipney H., Painter J.L., Shen J., Nicoletti P., Shen Y., Floratos A., Sham P.C., Li M.J., Wang J. (2015). The support of human genetic evidence for approved drug indications. Nat. Genet..

[68] King E.A., Davis J.W., Degner J.F. (2019). Are drug targets with genetic support twice as likely to be approved? Revised estimates of the impact of genetic support for drug mechanisms on the probability of drug approval. PLoS Genet..

[69] Minikel E.V., Painter J.L., Dong C.C., Nelson M.R. (2024). Refining the impact of genetic evidence on clinical success. Nature.

[70] Cook D., Brown D., Alexander R., March R., Morgan P., Satterthwaite G., Pangalos M.N. (2014). Lessons learned from the fate of AstraZeneca’s drug pipeline: A five-dimensional framework. Nat. Rev. Drug Discov..

[71] Morgan P., Brown D.G., Lennard S., Anderton M.J., Barrett J.C., Eriksson U., Fidock M., Hamrén B., Johnson A., March R.E. (2018). Impact of a five-dimensional framework on R&D productivity at AstraZeneca. Nat. Rev. Drug Discov..

[72] Lee C.A., Schreiber S., Bressler B., Adams J.W., Oh D.A., Tang Y.Q., Zhang J., Komori H.K., Grundy J.S. (2024). Safety, pharmacokinetics, and pharmacodynamics of etrasimod: Single and multiple ascending dose studies in healthy adults. Clin. Pharmacol. Drug Dev..

[73] Argente J., Verge C.F., Okorie U., Fennoy I., Kelsey M.M., Cokkinias C., Scimia C., Lee H.M., Farooqi I.S. (2025). Setmelanotide in patients aged 2–5 years with rare MC4R pathway-associated obesity (Venture): A 1-year, open-label, multicentre, phase 3 trial. Lancet Diabetes Endocrinol..

[74] Shi L., Lehto S.G., Zhu D.X.D., Sun H., Zhang J., Smith B.P., Immke D.C., Wild K.D., Xu C. (2016). Pharmacologic characterization of AMG 334, a potent and selective human monoclonal antibody against the calcitonin gene-related peptide receptor. J. Pharmacol. Exp. Ther..

