# Supplementary material for: Clinically Anchored GPCR Drug Discovery: Translational Lessons from Success, Failure, and Post-Approval Reassessment
Source: Pharmaceuticals (Basel). 2026 Sep 23;19(10):1510. doi: 10.3390/ph19101510 (PMC13708073; doi:10.3390/ph19101510)
Supplement: Supplementary file 1 [file pharmaceuticals-19-01510-s001.zip › pharmaceuticals-4564983-supplementary.pdf]

Supplementary Table S1

Expanded operational definitions for the five-axis GPCR translational scorecard

Axis (weight)	Score 0	Score 1	Score 2	Stage-specific hard-stop / interpretation
A1. Human-disease anchoring (25%)	No direct human support sufficient to link the receptor to the disease mechanism or define a responsive population.	Human association or biologic plausibility is present, but causal or population-defining evidence is incomplete or heterogeneous.	Causal genetic, anatomic, or human perturbation evidence defines the disease mechanism and the population in which the target is expected to matter.	A1 = 0 at target nomination: do not nominate without new causal human evidence.
A2. Receptor pharmacology (15%)	Subtype selectivity, redundancy, signaling, or tissue logic is unresolved to the point that receptor engagement cannot be interpreted reliably.	Core pharmacology is partly characterized, but clinically relevant redundancy, subtype effects, signaling context, or tissue dependence remains uncertain.	Selectivity, endogenous-ligand/signaling behavior, tissue logic, and relevant redundancy are sufficiently defined for the intended indication and modality.	No automatic hard stop; a low score identifies a receptor-pharmacology experiment that must be resolved before escalation.
A3. Modality and PK/PD feasibility (20%)	Required exposure or target engagement is clinically infeasible, unacceptably unsafe, or incompatible with the intended route/reversibility needs.	Clinically feasible in principle but constrained by route, exposure duration, drug–drug interactions, reversibility, tissue access, or therapeutic-window limitations.	Exposure, route, reversibility, tissue access, and target-engagement control fit the indication with a clinically manageable PK/PD strategy.	A3 = 0 before clinical advancement: do not advance until the modality/exposure strategy is redesigned.
A4. Biomarker-to-endpoint readiness (25%)	No mechanism-proximal target-engagement/PD readout, or available biomarker is disconnected from the intended clinical endpoint.	Target engagement or PD can be measured, but linkage to the registration endpoint and/or responsive-patient selection remains uncertain.	Mechanism-proximal PD/target engagement is measurable and credibly linked to the clinical endpoint with an appropriate patient-selection strategy.	A4 = 0 before late-stage commitment: stop or redesign the biomarker/endpoint strategy.
A5. Durability (15%): lower of 5A safety and 5B evidence reliability	5A: unacceptable toxicity/benefit–risk. 5B: pivotal evidence is unreliable or not decision-grade.	5A: safety risk is uncertain but monitorable/mitigable. 5B: evidence is credible but incomplete, fragile, or requires confirmation.	5A: safety is acceptable for the intended exposure with a manageable monitoring plan. 5B: efficacy evidence is credible, reproducible, and decision-ready.	At registration/post-approval, 5A = 0 or 5B = 0 triggers hold, withdrawal, or independent evidence regeneration irrespective of total score.

Scoring formula and NA rule. When all five axes are assessable, weighted total = Σ (axis score ÷ 2) × axis weight. A wholly non-assessable (NA) axis is excluded from both the weighted numerator and the assessable-weight denominator, and the total is normalized to the remaining assessable weight. If one Axis 5 component is genuinely NA, the provisional Axis 5 score is based on the assessable component and reported as x/NA; once both components are assessable, Axis 5 is the lower component score. Evidence that could reasonably have been generated at the relevant stage but was not measured is scored 0 rather than NA.

Decision bands. ≥75: advance; 60–74: conditional/rework; <60: stop/rework. Any stage-specific hard stop overrides the numerical total. The weights and thresholds are reasoned reporting conventions rather than empirically fitted coefficients and require prospective external validation.
